# Methicillin-Resistant *Staphylococcus aureus* USA300 Clone in Long-Term Care Facility 

**DOI:** 10.3201/eid1506.080195

**Published:** 2009-06

**Authors:** Pierre Tattevin, Binh An Diep, Michael Jula, Françoise Perdreau-Remington

**Affiliations:** Pontchaillou University Hospital, Rennes, France (P. Tattevin); San Francisco General Hospital, San Francisco, California, USA (B.A. Diep, M. Jula, F. Perdreau-Remington); 1 Current affiliation: San Francisco General Hospital, San Francisco, California, USA.

**Keywords:** Staphylococcus aureus, staphylococci, bacteria, antimicrobial resistance, long-term care facility, epidemiology, USA300, California, USA, dispatch

## Abstract

We performed a longitudinal analysis of 661 methicillin-resistant *Staphylococcus aureus* (MRSA) isolates obtained from patients in a long-term care facility. USA300 clone increased from 11.3% of all MRSA isolates in 2002 to 64.0% in 2006 (p<0.0001) and was mostly recovered from skin or skin structures (64.3% vs. 27.0% for non-USA300 MRSA; p<0.0001).

Since 2001, a dramatic increase in methicillin-resistant *Staphylococcus aureus* (MRSA) infections has been observed in the United States, mostly related to emergence of the USA300 clone in the community (*1*) and subsequently in hospitals (*2*,*3*). Residents of long-term care facilities (LTCFs) are at risk for colonization with antimicrobial drug–resistant bacteria, including MRSA. After they have been colonized, these residents are at increased risk for infections (*4*). Although it is assumed that transfer of patients between acute-care hospitals and LTCFs provides an ongoing cycle for the introduction of MRSA between these facilities (*5*), few studies have described the molecular epidemiology of MRSA in LTCFs. We report the prevalence and distribution of MRSA genotypes among clinical isolates obtained over a 10-year period (1997–2006) at the main LTCF in San Francisco, California, USA.

## The Study

The San Francisco Laguna Honda Hospital is a 1,000-bed LTCF. We conducted a retrospective review of electronic records for all cultures positive for *S*. *aureus* that originated from hospital residents during 1997–2006. Colonization screening was not performed during the study period. Data collection was approved by the Committee on Human Research, Office of Research Administration, at the University of California, San Francisco.

Isolates were tested for oxacillin resistance by the salt agar method, and the presence of the *mec*A gene was confirmed by PCR. Susceptibility to other antimicrobial drugs was determined by using microbroth dilution with the MicroScan WalkAway 96 instrument (Dade Behring, Deerfield, IL, USA). Inducible clindamycin-resistance testing (D-zone test) was performed by using the agar disk-diffusion method for isolates obtained during 2005–2006. Results were interpreted in accordance with guidelines (M7-A5) of the Clinical and Laboratory Standards Institute (Wayne, PA, USA; www.clsi.org).

Nonduplicated MRSA isolates were genotyped by pulsed-field gel electrophoresis (PFGE) after digestion of chromosomal DNA with *Sma*I (*6*), *spa* typing (*7*), and multilocus sequence typing (MLST) (*8*). USA300 was defined by the presence of Panton-Valentine leukocidin (PVL) genes (*luk*F-PV and *luk*S-PV) and the arginine catabolic mobile element (ACME), detected by PCR. Staphylococcal cassette chromosome *mec* (SCC*mec*) type was identified by using a PCR-based protocol (*9*). All strains underwent *spa* typing and were tested for PVL and ACME genes. MLST was conducted for 12 strains that could not be characterized otherwise.

Chi-square tests were used for bivariate analysis, and χ^2^ tests for trend were used to evaluate secular trends. All statistical analysis was conducted by using Stata version 9.1 (Stata Corp., College Station, TX, USA).

**Figure 1:**
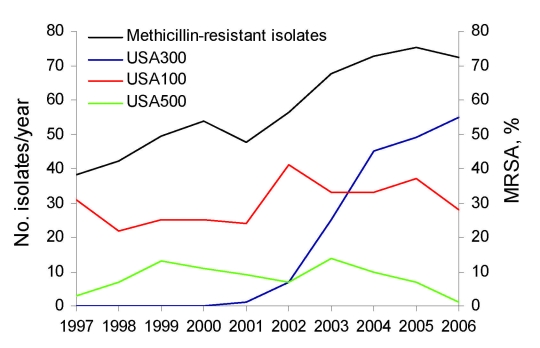
Longitudinal dynamics of methicillin resistance and methicillin-resistant *Staphylococcus aureus* (MRSA) clones at a long-term care facility, San Francisco, California, USA, 1997–2006.

**Figure 2:**
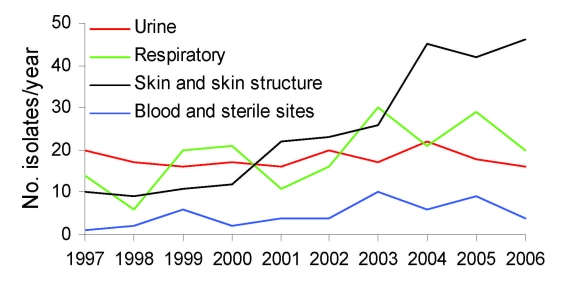
Evolution of methicillin-resistant *Staphylococcus aureus* isolate specimen sources at a long-term care facility, San Francisco, California, USA, 1997–2006.

*S*. *aureus* was isolated from 1,284 patients. Of these isolates, 744 (57.9%) were MRSA. The proportion of MRSA among *S*. *aureus* isolates increased from 38.1% (56/147) in 1997 to 72.3% (99/137) in 2006 (p<0.0001; Figure 1). Median age (interquartile range [IQR]) was 67 years (54–78 years) for patients with MRSA and 64 years (49–78 years) for those with methicillin-susceptible *S*. *aureus* (MSSA) (p = 0.22). Male:female ratio was 1:2.2 for MRSA and 1:1.7 for MSSA (p = 0.12). For MRSA specimen sources, we observed a decrease in the proportion of urinary or respiratory specimens from 69.7% (131/188) during 1997–2000 to 49.5% (236/477) during 2001–2006, and a concomitant increase in the proportion of skin or skin structure specimens from 22.3% (42/188) to 42.8% (204/477) (p<0.0001; Figure 2). No change in specimen sources was observed for MSSA.

Three PFGE clonal groups (USA100 [ST5, SCC*mec* type II], USA300 [ST8, SCC*mec* type IV], and USA500 [ST8, SCC*mec* type IV]) accounted for 85.2% (563/661) of genotyped MRSA isolates. USA300 was first isolated in 2001 and accounted for 11.3% of all MRSA (7/62) in 2002, 30.1% (25/83) in 2003, 47.9% (45/94) in 2004, 49.5% (49/99) in 2005, and 64.0% (55/86) in 2006 (p<0.0001; Figure 1). Compared with non-USA300 MRSA, USA300 was most frequently isolated from skin or skin structures (64.3% vs. 27.0%, risk ratio 2.38, 95% confidence interval 1.98–2.87, p<0.0001) and was recovered from younger patients with a median age (IQR) of 62 years (51–75 years) vs. 68 years (54–79 years; p = 0.001).

Among all MRSA isolates, gentamicin resistance decreased from 46.9% in 1997 to 4.7% in 2006, and clindamycin resistance decreased from 71.4% to 47.6% for the same years (p<0.0001). Overall, 285/744 (38.3%) MRSA isolates were multidrug resistant (resistant to >3 non–β-lactam antimicrobial drugs), including 177/299 (59.2%) USA100, 39/182 (21.4%) USA300, and 32/82 (39.0%) USA500. Inducible clindamycin resistance was detected in 15/281 (5.3%) MRSA isolates during 2005–2006. The rate of multidrug resistance for USA300 was lower than for non-USA300 (p<0.0001) but increased from 0% in 2001 to 14.3% (1/7) in 2002, 12.0% (3/25) in 2003, 13.3% (6/45) in 2004, 24.5% (12/49) in 2005, and 30.9% (17/55) in 2006 (p = 0.015). USA300 was less frequently resistant than non-USA300 to clindamycin (p<0.0001), gentamicin (p = 0.0004), and trimethoprim/sulfamethoxazole (p = 0.0001) and more frequently resistant to tetracycline (p = 0.0002) (Table). Resistance to vancomycin, linezolid, dalbavancin, and daptomycin was not detected.

**Table:** Analysis of MRSA clones obtained in a long-term care facility, San Francisco, California, USA, 1997–2006*

Clonal group	Antimicrobial drug resistance, %							Specimen source, %			
	Ery	Cli	Cip	Sxt	Gen	Tet		SST	Respiratory tract	Urine	Blood†
USA100 (n = 299)	96.2	84.9	95.9	3.3	16.9	2.6		22.7	36.4	35.1	5.8
USA300 (n = 182)	87.8	15.0	79.4	0.6	6.1	13.2		64.3	18.1	10.4	7.1
USA500 (n = 82)	84.7	27.8	71.7	28.2	20.8	6.0		30.5	28	31.7	9.8
LFT (n = 98)	87.3	65.6	62.8	10.9	13.4	7.8		37.2	26.6	26.6	9.6
All MRSA (n = 744)	91.8	64.6	87.0	7.1	21.6	5.1		32.7	25.1	23.1	6.3

*MRSA, methicillin-resistant *Staphylococcus aureus*; Ery, erythromycin; Cli, clindamycin; Cip, ciprofloxacin; Sxt, trimethoprim-sulfamethozazole; Gen, gentamicin; Tet, tetracycline; SST, skin and skin structure; LFT, low frequency types. †Blood and other typically sterile sites.

## Conclusions

Increasing incidence of MRSA infections in this LTCF during 1997–2006 is attributable to 2 clonal groups; USA100 predominated until 2003, and USA300 predominated during 20042006. Emergence of a new MRSA clone in healthcare facilities may be followed by a decrease in incidence of other MRSA clones (*10*). Unfortunately, this decrease was not observed in this LTCF, where the incidence of the previously predominant clone USA100 remained unabated. As a consequence, emergence of USA300 led to a 2-fold increase in MRSA incidence and an increase to 73% in the rate of methicillin resistance among *S*. *aureus*. Moreover, because USA300 has a tropism for skin and skin structure infections (*3*), its emergence caused a shift in MRSA specimen sources.

Although primarily encountered in urinary and respiratory specimens until 2001 in this study, as in previous studies performed in LTCFs, MRSA isolates have mostly originated from skin or skin structure since 2002. This finding has important implications for MRSA transmission and should be taken into account when designing infection control policies in LTCFs. For example, nasal decolonization as a means to prevent MRSA infection implies that MRSA reservoirs reside in endogenous sources. However, skin–skin and skin–fomite contact may represent common alternative routes of transmission for USA300 (*11*).

In contrast to reports characterizing USA300 strains as typically not multidrug resistant (*1*), we found that up to 30.9% of USA300 isolates were multidrug resistant. This finding suggests that USA300 isolates were acquired under antimicrobial drug pressure in the LTCF or during a stay in another hospital rather than while in contact with the community. Results from this study and others illustrate the fitness trait of USA300 clonal lineage (*12*). To prevent further spread of multidrug-resistant USA300 in LTCFs would require enhanced infection control policies, which may include isolation or cohorting of infected patients, antimicrobial drug stewardship, and systematic use of alcohol-based handwashing products. However, given that these policies have proven difficult to implement in tertiary care hospitals, it may be even more challenging in LTCFs, which have limited staff and infection control resources (*5*,*13*–*15*).

This study had some limitations. Increased incidence of MRSA-positive cultures could be related to changes in sampling policies in this institution (e.g., more frequent sampling, surveillance cultures). However, the increased incidence rate for MRSA is not likely related to these changes because the annual number of cultures positive for any pathogen gradually decreased over the study period, from 1,433 in 1997 to 862 in 2006, as did the incidence of MSSA-positive cultures, from 91 in 1997 to 38 in 2006.

Although our study was limited by its reliance on retrospective data collection, it illustrates the need for further investigations in LTCFs regarding risk factors and appropriate interventions to minimize further transmission of MRSA. LTCFs, long thought to be reservoirs of nosocomial MRSA clones, now emerge as an important reservoir for USA300 and could play a role in the emergence of multidrug-resistant USA300.
